# Pelagic Shuttles of Antibiotic Resistance Genes: Zooplankton as Overlooked Vectors Across Space and Food Webs

**DOI:** 10.1007/s00248-025-02669-z

**Published:** 2025-11-25

**Authors:** Albert Calbet

**Affiliations:** Institut de Ciències del Mar (ICM-CSIC), Passeig Marítim de la Barceloneta 37–49, 08003 Barcelona, Spain

**Keywords:** Antibiotic resistance genes (ARGs), Mobile genetic elements (MGEs), Zooplankton, Phagotrophic protists, Horizontal gene transfer (HGT), One health

## Abstract

Antibiotic resistance genes (ARGs) accumulate in aquatic environments, where they create reservoirs and transmission pathways that can undermine antimicrobial treatments and alter the microbial community structure in ways that ultimately affect human and animal health. However, the contribution of zooplankton in these pathways remains critically overlooked. Emerging evidence shows that compared with surrounding water, copepods and cladocerans accumulate ARG loads that are one to two orders of magnitude greater, acting as microbial hotspots that disperse resistant bacteria across seasons and depths. Inside protistan vacuoles, densely packed prey cells undergo conjugation, rapidly accelerating horizontal ARG transfer. Long-term archives reveal persistent ocean-wide dissemination of the class-1 integron integrase (intI1) and sul2 genes since at least the 1970s. Here, I synthesize mechanistic and field evidence, pinpoint knowledge gaps, and recommend priorities: integrate zooplankton into routine ARG surveillance, quantify biofilm-mediated exchanges, and mitigate contamination from coselective pollutants to curb zooplankton-driven ARG propagation. By framing zooplankton-associated ARG dynamics within the broader community ecology of antimicrobial resistance, this mini-review highlights how aquatic food-web processes feed back into the emergence, evolution, and transmission of resistance that concerns for One Health outcomes beyond the clinic.

## Introduction

Antibiotic resistance genes (ARGs) now rank among the most pervasive anthropogenic contaminants, with an estimated 1.27 million deaths in 2019 indirectly attributable to bacterial antimicrobial resistance in clinical settings [1]. On their own, resistance traits do not cause disease or mortality; they become a public health concern when they are acquired by pathogenic bacteria and compromise the efficacy of available drugs. Environmental microbiomes act as reservoirs and evolutionary arenas for ARGs and mobile genetic elements that can eventually move into human and animal pathogens [2–5]. Among these environments, aquatic systems are especially crucial because municipal, agricultural, and industrial effluents enter these systems, and these aquatic systems act as long-distance conveyors of microbial genetic material [2, 3].

Within the pelagic food web, zooplankton occupy a central trophic position and create high-density microenvironments—exoskeletons, feeding currents, guts, and sinking fecal pellets—that favor intense microbe–microbe interactions. Copepods, for example, host bacteria in abundances up to 10³-fold higher than ambient water and have therefore been described as “microbial hotspots” [6]. Freshwater cladocerans such as *Daphnia* can protect wastewater-derived resistant bacteria from predation, UV stress, and other environmental threats, effectively serving as mobile refuges for ARGs [7].

The role of zooplankton in shaping ARG biogeography remains largely overlooked in current surveillance programs, which tend to focus on free-living microbes or benthic sediments. One plausible reason is the long-standing assumption that protistan phagotrophs (encompassing heterotrophic and mixotrophic unicellular protists, including ciliates, flagellates, and amoebae) capture and digest their prey efficiently so that food vacuoles and guts act as ecological dead ends for ingested bacteria and their genes. However, recent work has shown that a fraction of prey cells and transconjugants can survive gut passage or be expelled and that extensive lysis within vacuoles releases extracellular DNA that can be assimilated by other bacteria, indicating that these compartments can contribute to ARG turnover at the community level [8–10]. These findings indicate the requirement for an integrated One Health approach that explicitly incorporates both proto- and metazooplankton into risk assessments and mitigation frameworks, recognizing that the health of humans, domestic animals, crops, and ecosystems is tightly linked and that environmental selection and transmission of resistance can feed back into clinical risk [4, 5].

Aquatic zooplankton generate a mosaic of microhabitats whose physicochemical properties diverge sharply from those of the surrounding bulk water. At the micrometer–millimeter scale, these niches concentrate bacteria, viruses, and mobile genetic elements, creating conditions that intensify horizontal gene transfer (HGT) and, in turn, support the persistence and spread of ARGs. Metazoan substrates—chitinous copepod carapaces and gelatinous larvacean “houses”—trap organic particles and exude sticky polysaccharides; the bacterial loads on these surfaces typically exceed those of ambient seawater by orders of magnitude, making them microbial hotspots [11–13]. Within phagotrophic protists, food vacuoles function as extreme microreactors: when donor and recipient bacteria are compressed into these compartments, plasmid transfer can increase over short time frames, including for conjugative systems such as RP4 [8, 14, 15]. Crustacean guts and their rapidly sinking fecal pellets serve as vertical conveyors for dense, bacteria-rich communities across oxic–anoxic gradients, which is consistent with extensive evidence that pellets and zooplankton surfaces are sites of elevated microbial abundance and activity [16–18]. ARG markers have been detected in plankton-associated fractions, including long-term Continuous Plankton Recorder (CPR) archives for intI1 and sul2 [19] and regional surveys reporting tetA, bla variants, sul1, and intI1 in phyto- and zooplankton [20]. To the best of my knowledge, ARGs have not yet been quantified specifically inside marine zooplankton fecal pellets—an open, testable knowledge gap.

Several nonexclusive processes explain how these hotspots yield outsized ARG signals. First, protistan grazing physically disrupts prey cells, releasing extracellular DNA that can be assimilated by competent bacteria; controlled cocultures of algae and ciliates show rapid increases in dissolved DNA and elevated natural transformation frequencies within hours [8]. Second, ciliate vacuoles provide mildly anoxic yet ROS (reactive oxygen species)-rich microenvironments that can accelerate conjugation; laboratory syntheses report order-of-magnitude increases for clinically relevant β-lactamase and tetracycline plasmids under vacuole-like conditions versus free-living controls [8]. Third, antagonistic interactions involving antibiotic-producing bacteria and their neighbors can coenrich biosynthesis-related gene clusters and parallel resistance determinants, which is consistent with the “weapons-and-shields” paradigm, wherein producers require self-resistance [21, 22]. Finally, sublethal grazing cues can upregulate adhesion and promote biofilm formation on biotic substrates—including zooplankton exoskeletons, fecal pellets, and intestinal linings—bringing cells into intimate contact and enhancing both conjugation and transformation [23]. In the sections that follow, I first synthesize evidence that phagotrophic protist vacuoles can act as microreactors that accelerate horizontal gene transfer; I then examine how metazooplankton such as cladocerans and copepods serve as mobile reservoirs and vectors across trophic levels and depth gradients; and finally, I discuss how environmental drivers and surveillance strategies shape the One Health implications of these processes.

## Phagotrophic Protists: Microreactors for Horizontal Gene Transfer

Phagotrophic protists produce tightly packed digestive or phagocytic vacuoles in which donor and recipient bacteria are compressed to densities exceeding 10⁹ cells mL⁻¹. Although these vacuoles are primarily digestive, and most ingested biomass is degraded, a nonzero fraction of prey cells and newly formed transconjugants can persist long enough to be expelled in egesta or after protist death, and extensive lysis within vacuoles releases extracellular DNA that can be assimilated by competent bacteria. Thus, even compartments long regarded as ecological dead ends can contribute to ARG turnover at the community level. The conjugative plasmid RP4 moves from Escherichia coli to diverse γ-proteobacterial recipients within *Tetrahymena pyriformis* vacuoles at frequencies one to two orders of magnitude above those in the surrounding medium [14, 15]. The transfer of blaNDM-5 from *E. coli* to other clinically relevant pathogens has been demonstrated within ciliate vacuoles, with transconjugants recovered after short incubation periods [24]; similar phenomena have been documented for β-lactamase- and fluoroquinolone-resistance plasmids, with single-event transfer rates reaching 10⁻⁵ per donor in ciliate vacuoles [24, 25].

Amoebae provide a parallel but more selective niche: while Acanthamoeba vacuoles allow Legionella to survive and occasionally exchange effector genes [9], evidence for consistent plasmid-borne ARG transfer in Acanthamoeba is scarce and context dependent, underscoring that the HGT potential varies among protist lineages and even among particular amoeba–bacterium combinations [8, 10].

Environmental context modulates these processes. Under anaerobic or low-oxygen conditions—common in wastewater biofilms—digestion is slow in protists, and this lengthens the window during which conjugation can occur; low-redox wastewater conditions increase conjugative transfer frequencies by nearly an order of magnitude relative to those of aerobic controls [26]. Oxidative stress can stimulate the bacterial SOS response, upregulating the conjugation machinery and enhancing HGT: disinfection with subinhibitory concentrations of chlorine elevated intracellular ROS in *Acinetobacter baylyi* and increased natural transformation frequencies, with effects suppressed by ROS scavengers or under anaerobic conditions [27]. Low concentrations of heavy metals such as copper and silver can coinduce the ROS and SOS pathways in E. coli, significantly increasing the conjugative transfer of multiresistant plasmids [28]. Conversely, when protists actively graze on biofilms, they can suppress ARG dissemination by disrupting cell-to-cell contacts [23], underscoring the dual—either magnifying or mitigating—role of phagotrophic protists in resistome dynamics.

## Metazooplankton: Mobile Reservoirs and Vectors Across the Food Web

**Fig. 1:**
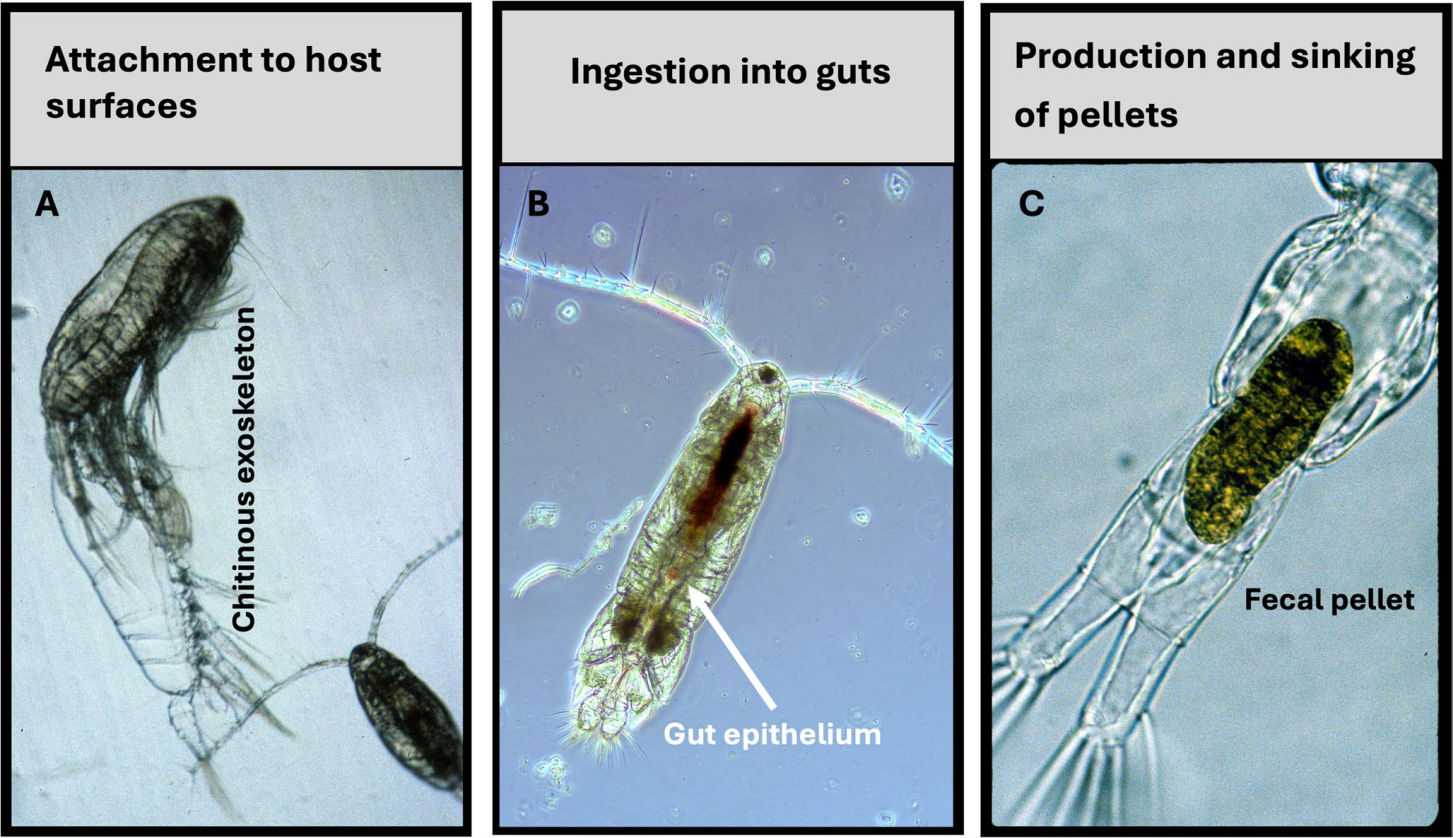
Mesozooplankton-associated hotspots of antibiotic resistance gene (ARG) exchange. (**A**) Chitinous exoskeletons support dense cuticular biofilms where bacteria, phages, and mobile genetic elements accumulate. (**B**) The gut epithelium and lumen host concentrated, often anoxic communities in which conjugation and other HGT processes can be enhanced. (**C**) Rapidly formed fecal pellets package bacteria and ARGs into sinking particles that transport resistance vertically and laterally through the water column

Crustacean zooplankton concentrate bacteria and their ARGs on three principal substrates: chitinous exoskeletons, gut epithelium, and rapidly sinking fecal pellets (Fig. 1). A 41-year transect of CPR samples revealed that copepod-rich tows contained the class-1 integrase intI1 and the sulfonamide marker sul2 in 76% and 48% of samples, respectively [19]. In the Seine estuary, compared with free-living isolates, Aeromonas populations attached to calanoid copepods presented distinct genotypes and phenotypic resistance patterns, indicating that host surfaces act as refuges where selection and gene exchange can cooccur [29]. Enrichment within zooplankton does not represent an ecological dead end. A controlled mesocosm linking manure-amended pond water to a *Daphnia magna* → bighead carp (Aristichthys nobilis) food chain revealed the stepwise bioaccumulation of 14 ARGs; the concentrations of four loci (tetM-01, tetX, qnrS, sul2) increased significantly from the water to the daphnid gut and again to the fish gut, while cooccurrence networks identified intI1/intI2 as hubs in transfer [30]. Mechanistic evidence comes from gut-passage conjugation assays: within four hours of feeding, vancomycin-resistant Enterococcus faecalis donors transferred the vanA operon to rifampicin-tagged recipients inside *D. magna* and *D. pulex* intestines, generating dual-resistant transconjugants [31]. Together, these studies have demonstrated that metazooplankton such as cladocerans and copepods can substantially increase the prevalence of multidrug-resistant bacteria within commercially important fish via their food-web interactions, thus acting as active vectors rather than passive carriers of resistance genes.

The ecological impact of metazooplankton-associated ARGs depends not only on host traits but also on behavior and physicochemical context. Diel vertical migration redistributes attached microbial consortia—and their associated resistomes—through tens to hundreds of meters of the water column, while the production and sinking of fecal pellet strings delivers dense bacterial assemblages into deeper, often anoxic, layers [17, 18]. These processes provide a conduit for ARGs to cross otherwise stable redox boundaries and seed mesopelagic communities. Along with this physical transport, the local abundance of mobile genetic elements and ambient stressors (e.g., metals, disinfectants, hypoxia) likely modulate resistome composition and HGT rates, helping explain the spatial heterogeneity observed in plankton-associated ARG signals [19, 20, 23].

## Discussion and Outlook

The emerging literature converges on a unifying framework: phagotrophic protists act as biochemical reactors that intensify HGT, whereas metazooplankton function as physical shuttles that accumulate, protect, and disseminate ARGs across trophic levels and along hydrological corridors. This dual role is supported by controlled laboratory experiments [14], mesocosm food chain set-ups [30], and long-term oceanic archives [19].

Whether zooplankton ultimately mitigate or magnify resistance depends on the net ecological outcome of their interactions with microbes. Grazing can reduce pathogen abundance by disrupting biofilms and contact networks and lowering HGT rates [23]; conversely, digestive vacuoles and anoxic gut environments can create hotspots for conjugative transfer, often increasing HGT frequencies relative to those in bulk water [26]. Environmental stressors—including temperature anomalies, trace metals, and sublethal disinfectant exposure—can further modify this balance by enhancing the stability and exchange of MGEs [27, 28].

From a One Health perspective, incorporating zooplankton into ARG surveillance is a powerful and increasingly feasible extension of existing monitoring frameworks rather than a purely academic refinement. Filter-feeding zooplankton concentrate bacterial biomass and associated contaminants by orders of magnitude relative to those of ambient water, effectively functioning as sentinel species. Routine plankton net sampling, coupled with quantitative PCR (qPCR), droplet digital PCR (ddPCR) where available, or targeted metagenomics, can be integrated into coastal monitoring programs and ballast-water inspections, with sentinel markers such as intI1 and sul2 quantified in zooplankton-associated fractions rather than only in bulk water [19, 32]. Because zooplankton can harbor wastewater-derived resistant bacteria and coselective pollutants before these bacteria and pollutants reach problematic thresholds in surrounding water or fish tissue, such measurements would provide early warning of elevated ARG loads and of “time-bomb” exposure scenarios that are not discernable from water chemistry alone. As qPCR infrastructure is already available in many water-quality laboratories, the main hurdles are the analytical capacity for zooplankton-associated samples and the regulatory recognition of zooplankton as sentinels; both can be addressed gradually by piloting low-cost qPCR assays and incorporating zooplankton metrics into existing AMR reporting frameworks. Mitigation efforts should prioritize the control of coselective pollutants—metals, biocides, and microplastics—that amplify resistance selection and stabilize ARGs on zooplankton-associated surfaces, and wastewater processes should be engineered to disrupt anaerobic conjugation hotspots [26].

A major knowledge gap concerns viral and vesicle-borne ARGs within zooplankton-associated niches. Phages, plasmid–phage hybrids, and extracellular vesicles are being increasingly recognized as drivers of ARG dissemination [33, 34]. However, their quantitative role within copepod guts, particle-attached biofilms, and sinking fecal pellets remains largely unexplored.

Taken together, three conservative, data-anchored lines of evidence indicate material risk. First, the prevalence at the source is high: 63%–88% of phyto- and zooplankton samples in the English Channel and North Sea carry intI1 and/or sul1 [20], and CPR archives have detected intI1 in 76% and sul2 in 48% of historical plankton tows [19]. Second, trophic transfer has been demonstrated experimentally: in mesocosms, the abundance of 14 ARGs increased stepwise from water to Daphnia guts and then to fish intestines, with intI1/intI2 acting as network hubs [30], and vanA conjugation occurred within hours in daphnid intestines [31]. Third, exposure interfaces are substantial: ballast tanks and harbor sediments can harbor up to 10⁸–10⁹ copies g⁻¹ of sul1, often with intI1 exceeding sul-class markers, and ballast discharge elevates ARG loads in receiving waters [32, 35, 36], while aquaculture-relevant pathogens such as Aeromonas, Pseudomonas, and Vibrio frequently carry multidrug resistance genes [37, 38]. In addition to these reservoirs, migrating mesozooplankton biomass provides continuous throughput—globally, mesozooplankton produce ~ 4.2 Gt C yr⁻¹ in biomass and ~ 6.2 Gt C yr⁻¹ in fecal pellets [18, 39], so even without a knowledge of the precise ARG copies per pellet, the dynamics of trophic transfer and rapid gut-passage conjugation highlight the need for prioritized surveillance at aquaculture sites exposed to ballast traffic, effluents, or warming-induced hypoxia.

In conclusion, zooplankton-mediated ARG dissemination represents a credible, quantifiable One Health risk. Mitigation requires (i) incorporating zooplankton into ARG monitoring networks; (ii) regulating coselective pollutants in aquaculture and port environments; and (iii) investing in research that disentangles conjugative, viral, and vesicle-mediated pathways. Stronger integration of ecological and clinical surveillance will be essential for translating mechanisms into management schemes.

## Data Availability

No datasets were generated or analysed during the current study.

## Declarations

### Use of AI‑assisted Technologies

During manuscript preparation, the author used ChatGPT for language and style editing, followed by professional proof-editing. The author reviewed and edited the content and takes full responsibility for the final text.
